# The three-dimensional easy morphological (3-DEMO) classification of scoliosis – Part III, correlation with clinical classification and parameters

**DOI:** 10.1186/1748-7161-2-5

**Published:** 2007-03-19

**Authors:** Stefano Negrini, Alberto Negrini

**Affiliations:** 1 ISICO (Italian Scientific Spine Institute), Milan and Fondazione Don Carlo Gnocchi IRCCS-ONLUS, Milan, Italy

## Abstract

**Background:**

In the first part of this study we proposed a new classification approach for spinal deformities (3-DEMO classification). To be valid, a classification needs to describe adequately the phenomenon considered (construct validity): a way to verify this issue is comparison with already existing classifications (concurrent and criterion validity).

**Aim:**

To compare the 3-DEMO classification and the numerical results of its classificatory parameters with the existing clinical classifications and the Cobb degrees on the frontal and sagittal planes respectively.

**Methods:**

118 subjects (96 females) with adolescent idiopathic scoliosis (age 15.9 ± 3.1, 37.4 ± 12.5° Cobb) have been classified according to 3-DEMO, SRS-Ponseti, King and Lenke classifications as well as according to sagittal configuration. For all patients we computed the values of the 3-DEMO parameters and the classical Cobb degrees measurements in the frontal and sagittal planes. Statistical analysis comprised Chi Square and Regression analysis, including a multivariate stepwise regression.

**Results:**

Three of the four 3-DEMO parameters (Direction, Sagittal and Frontal Shift) correlated with SRS-Ponseti, King and sagittal configuration classifications, but not with Lenke's one. Feeble correlations have been found among numerical parameters, while the stepwise regression allowed us to develop almost satisfactory models to obtain 3-DEMO parameters from classical Cobb degrees measurements.

**Discussion:**

These results support the hypothesis of a possible clinical significance of the 3-DEMO classification, even if follow-up studies are needed to better understand these possible correlations and ultimately the classification usefulness. The most interesting 3D parameters appear to be Direction and mainly Phase, the latter being not at all correlated with currently existing classifications. Nevertheless, Shift cannot be easily appreciated on classical frontal and sagittal radiographs, even if it could presumably be calculated.

## Background

The first proposed classification for scoliosis relates to the location of the various curves according to the apex vertebra, and has been initially developed by Schulthess [1], refined by Ponseti [2] and confirmed by the terminology committee of the Scoliosis Research Society [3]. This classification undoubtedly is bi-dimensional and based on AP radiographs. Nevertheless, it served its scope of communication among specialists, and it probably is the most generally used classification even today, because of its simplicity based on pure morphology. With years, mainly for surgical purposes, two other main classifications have been developed, whose names were gathered from the first author of the related publication: King in 1983 [4], and Lenke in 2001 [5]. The first one was mainly developed to distinguish type II curves that, in case of surgery, require a shorter fusion area than the others[4,6]; the main problems of this classification were the relatively low intra- and inter-observer reliability [7,8], the fact of totally being bi-dimensional and almost confined to thoracic curves[8,9]. Lenke's classification is far more complex, being an advancement of King's one and including lumbar and sagittal modifiers too, that represent an attempt to three-dimensionally look at the spine [6]: reliability seems to be good [5,10,11], but it is still relatively new and further studies are needed. Validity is the ability of a scale to measure what it is intended to measure[12]. It has been suggested that, for instruments designed to classify individuals, the demonstration of repeatability (BIB precedente) [12] and validity may be sufficient to ensure usefulness [13]. When looking at a new scale and/or classification, there are several kinds of validity to be considered. In this study we investigate:

• Construct validity: the extent to which the classification accurately represents a construct (real clinical entity: in this case vertebral deformities) and produces an observation distinct from that produced by a measure of another construct: does 3-DEMO produces something different from 2-D classifications, but anyway inherent to 3-D deformities?

• Concurrent validity: a method of determining validity of a classification as the correlation with scores of other valid classifications: does 3-DEMO correlates with other classifications ?

• Criterion validity: the degree to which a classification correlates with other of the same construct: does 3-DEMO correlates with other 3-D classifications ?

In the future, comparing with other existing 3-D classifications, together with completing the Concurrent and Criterion validity study performed today, it will be investigated also:

• Content validity: the ability of the classification to adequately represent the content of the property that the investigator wishes to measure: does 3-DEMO really evaluate 3-D the spine ?

Future clinical studies will allow to study:

• Predictive validity: how well a classification predicts outcome in a different population from the one from which it was derived: is 3-DEMO useful to predict clinical results ?

• External validity: the extent to which the classification applies (or can be generalized) to persons, objects, settings, or times other than those that were the subject of study: is 3-DEMO applicable in other settings ?

Demonstration of the possibility of future applications in everyday settings with usual clinical instruments will allow to consider the:

• Ecological validity: the extent to which the classification developed in laboratory reflect real life conditions: is 3-DEMO applicable in real everyday clinical life ?

Finally, partially assessed trough peer review and comments collected from peers during meetings, as well as future application by others, there is:

• Face validity: the clinical sense of a classification: does 3-DEMO makes sense given current understanding of scoliosis ?

While presenting a new classification as the 3-DEMO [14], it is crucial to compare its results with the existing clinical classifications, in order to understand their possible correlations: a complete superimposition could mean that this new system does not add anything, while a complete difference, being the evaluated phenomenon the same (scoliosis) even from a different perspective, could suggest a very different approach among considered systems. Moreover, numerical results of the 3-DEMO parameters should somehow correlate with Cobb degrees, because 2-D measurements are a partial description of a 3-D behaviour that nevertheless should be better described by 3-DEMO parameters: the degree of correlation is interesting to be looked at, to better understand the newly introduced numerical data.

In part I [14] of this study we presented the development methodology of the new 3-DEMO classification of scoliosis, while in Part II we considered its repeatability [15]. The aim of this paper is now to compare the 3-DEMO classification, as well as numerical results of its classificatory parameters, with existing clinical classifications and Cobb degrees on the frontal and sagittal planes respectively.

## Materials and methods

### Population

We included in this study 118 subjects (96 females) affected by adolescent idiopathic scoliosis. Mean age was 15.9 ± 3.1, while weight and height were 50.9 ± 10.8 and 160.2 ± 10.8 respectively. Scoliosis curvature had an average of 37.4 ± 12.5° Cobb, kyphosis was 35.4 ± 13.1° and lordosis 47.7 ± 12° Cobb.

### Classifications and radiographic parameters

Data have been acquired with the AUSCAN system and obtained curves have been classified according to the 3-DEMO classification, as described in the first part [14]. We also classified the patients according to SRS-Ponseti [2], King [4] and Lenke [5] classifications. For all patients we computed the values of the 3-DEMO parameters [14], as well as the classical Cobb degrees measurements in frontal and sagittal planes. According to the sagittal radiographic Cobb degrees, we classified the spinal sagittal configuration of each patient as follows [6]:

 Hyperkyphosis: kyphosis of more than 50° Cobb (18 patients);

 Flat-Back: kyphosis of less than 20° Cobb (46 patients);

 Hyperlordosis: lordosis of more than 60° Cobb (55 patients);

 Hypolordosis: lordosis of less than 30° Cobb (no patients).

Finally, considering the fact that 3-DEMO classification aims at merging in one single 3-D representation classical radiographic parameters, for each patient we computed a Cobb and a Sagittal Index. This was simply done with a sum of the angles in each radiographic plane, considering positive a right curve and lordosis, and negative a left curve and kyphosis. So, a 30° thoracic right, 20° lumbar left scoliosis had a Cobb Index of +10° (+30° -20° = +10°), and a kyphosis of 60° with lordosis of 45° produced a Sagittal Index of -15° (-60° +45° = -15°).

### Statistical analysis

**Figure 1:**
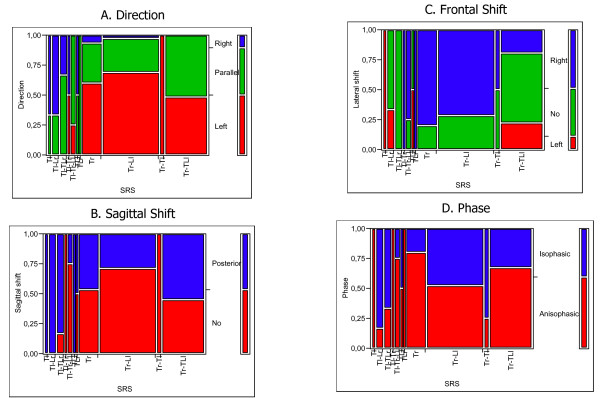
Correlation between the SRS-Ponseti classification and 3-DEMO parameters. In all graphs the legend of colors is reported in the vertical bar on the left (e.g. in graph A red means Left Direction, green is Parallel and blue Right Direction). In the graphs, each vertical bar reports the SRS classification, where in the abbreviations the high case letters means the spinal region involved (T: thoracic; TL: thoraco-lumbar; L: lumbar), while the low case letter means the side of convexity of the curve (r: right; l: left – e.g. Tr means Thoracic right, Ll means Lumbar left and so on). Moreover, all graphs both in abscissa and ordinate are scaled in percentage of cases. This means that the biggest vertical bar in the middle of graph A, with the abbreviation Tr-Ll, refers to Thoracic right Lumbar left scoliosis, i.e. the most numerous group of SRS classification, that has most of curves with Left Direction (red), and almost no Right Direction (blue) curves. A. The Direction 3-DEMO parameter more frequently represented is left; B. The Sagittal Shift 3-DEMO parameter never appear to be Anterior; C. The Frontal Shift 3-DEMO parameter is Right in over 50% of cases; D. The Phase parameter is equally distributed and is the only 3-DEMO parameter with no significant difference among SRS classified scoliosis.

All classifications have been compared with the 3-DEMO one using the Chi-square test. For the comparison between Ponseti and 3-DEMO classifications, after preliminary results were obtained, looking at the Figure 1, we grouped the patients according to the convexity of their thoracic curve (e.g. left Thoracic – right Thoraco-Lumbar, have been classified with the single left Thoracic and the left Thoracic – right Lumbar curves); in the group "Other" we included all the other type of curves, because their groups were small and the clinical meaning appear to be different. Variance analysis and regression line, with corresponding RSquare, have been calculated to correlate radiographic and 3-DEMO numerical parameters. Finally, we verified if the model offered by 3-DEMO numerical parameters was explainable by radiographic Cobb degrees through a stepwise multivariate regression analysis.

## Results

**Figure 2:**
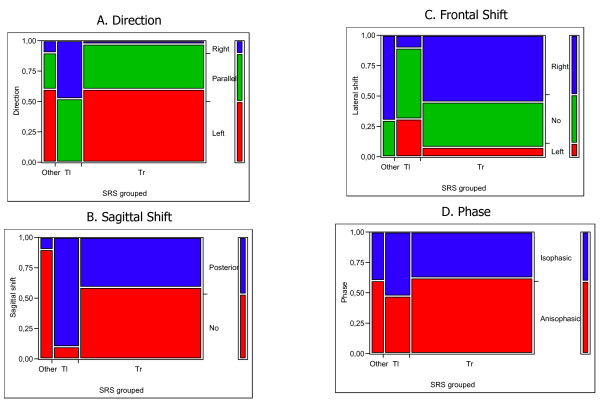
Correlation between 3-DEMO parameters and SRS-Ponseti classification, grouped according to the thoracic proximal curve: e.g. Moe's double curves (left proximal Thoracic – right distal Thoracic) have been classified with the single left Thoracic and the left Thoracic – right Lumbar curves, and so on. As explained in Figure 1, in all graphs the legend of colors is reported in the vertical bar on the left, each vertical bar reports the SRS classification (Tl: thoracic left; Tr: thoracic right), all graphs are scaled in percentage of cases. A. Left Direction is highly correlated with Thoracic right curves, while the opposite is true for Thoracic left; B. The Sagittal Shift 3-DEMO parameter is correlated with SRS classification, with Thoracic left curves not equally distributed, as it could have been supposed, being the SRS classification related to the frontal radiographs and not to sagittal ones; C. Right Frontal Shift prevails in Thoracic Right SRS curves, while this in Thoracic Left cases are almost equally distributed among the three possible Frontal Shift parameters; D. No differences have been seen according to the Phase 3-DEMO parameter.

3-DEMO parameters resulted statistically different among the groups according to the SRS classification, with the only exception of Phase (Figure 1): in particular, what appeared to drive these results is the localization of the thoracic curvae (Figure 2). On the contrary, looking at sagittal radiographic configuration, the only parameter that changed significantly among the groups was Sagittal Shift, that resulted posterior in case of associated Hypekyphosis, and not shifted when there was Flat Back alone or particularly with Hyperlordosis (Table 1).

**Table 1:** Correlation between Sagittal Shift 3-DEMO parameter and sagittal configuration in the considered scoliosis population.

	**Sagittal Shift**		
	**No**	**Posterior**	**Patients**

**Flat Back**	64.86%	35.14%	37
**Hyperkyphosis**	20.00%	80.00%	5
**Hyperkyphosis and Hyperlordosis**	38.46%	61.54%	13
**Hyperlordosis**	51.52%	48.48%	33
**Flat Back and Hyperlordosis**	88.89%	11.11%	9
**TOTAL**	56.70%	43.30%	97

**Figure 3:**
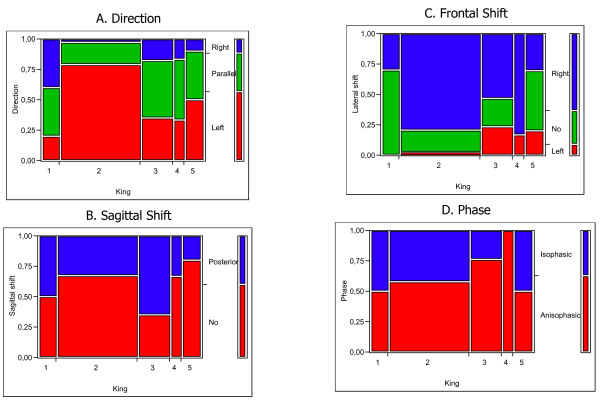
Correlation between King classification and 3-DEMO parameters. As explained in Figure 1, in all graphs the legend of colors is reported in the vertical bar on the left, each vertical bar reports the SRS classification (Tl: thoracic left; Tr: thoracic right), all graphs are scaled in percentage of cases. It can be easily seen that King curve type 2, typically highly represented, is correlated with Direction (graph A: left in 75% of cases) and Frontal Shift (graph C: right in over 75% of cases). Interestingly almost all King 4 curves were Anisophasic (graph D).

We did not find any correlation between 3-DEMO parameters and Lenke classification, even considering the modifiers. On the contrary, King classification was correlated with Direction and Lateral Shift: in particular, most of King 2 curves have Left Direction and Right Shift (Figure 3).

The correlation between 3-DEMO parameters and classical Cobb measurements (Table 2), even if statistically significant in many cases, was weak, with a dispersion of results around the predicted regression (low RSquare), the only exception being Cobb Index versus Lateral Shift with a RSquare of 0.35. The stepwise multivariate analysis allowed us to obtain better results, with a RSquare between 0.24 and 0.35 (Table 3): while Direction and Phase are determined by all radiographic parameters in both planes, showing to be totally 3-D, Shift data are characterized by sagittal (LL Shift) and frontal (AP-Shift, but also with the contribution of kyphosis) Cobb degrees values.

**Table 2:** Correlation between single 3-DEMO parameters and Cobb degrees in sagittal and frontal planes. Cobb and a Sagittal Index were obtrained with a sum of the angles in each radiographic plane, considering positive a right curve and lordosis, and negative a left curve and kyphosis.

	**3-DEMO parameters**							
	**Direction**		**LL Shift**		**AP Shift**		**Phase**	

	*RSquare*	*P*	*RSquare*	*P*	*RSquare*	*P*	*RSquare*	*P*

**Proximal Curve (°Cobb)**	0.08	0.003	0.07	0.004	0.11	<0.001	0.19	<0.001
**Distal Curve (°Cobb)**	0.01	NS	0.01	NS	0.04	0.04	0.15	<0.001
**Worst Curve (°Cobb)**	0.03	0.03	0.04	0.03	0.05	0.02	0.18	<0.001
**Cobb Index**	0.04	0.04	0.02	NS	0.35	<0.001	0.03	NS
**Kyphosis**	0.10	0.001	0.23	<0.001	0.05	0.02	0.03	NS
**Lordosis**	0.03	NS	0.00	NS	0.01	NS	0.00	NS
**Sagittal Index**	0.01	NS	0.15	<0.001	0.01	NS	0.02	NS

**Table 3:** Stepwise regression analysis: power of the best obtained model and related formula.

	**RSquare**	**P**	**Formula**				
			**Intercept**	**Proximal Cobb**	**Distal Cobb**	**Kyphosis**	**Lordosis**

**Direction**	0.28	<0.001	31.45	0,55	-0.50	-0.50	-0.03
**AP Shift**	0.36	<0.001	-0.61			0.59	-0.30
**LL Shift**	0.27	<0.001	6.90	0,25	-0.27	-0.11	
**Phase**	0.24	<0.001	4.37	0,08	0.09	-0.08	0.02

## Discussion

The 3-DEMO classification has already proven to be able to differentiate scoliosis patients from normals and to be repeatable [14,15]. In the process of validation of a new classification, ad unavoidable step is to verify if it describes adequately the phenomenon considered (construct validity): a way to verify this issue is the comparison with already existing classifications (concurrent and criterion validity). The best correlation between 3-DEMO and one of the other clinical existing classifications of idiopathic scoliosis has been found with Ponseti-SRS one. This can be easily understood when thinking that both classifications are morphological. Interestingly, the curve that seems to drive the 3-DEMO reconstruction is the thoracic one, as can be seen from Figure 1 and 2. In particular, right thoracic curves have a prevalence of left direction that corresponds to the direction of vertebrae rotation, right shift that corresponds to curve convexity, and no sagittal shift.

Type 2 curves have been considered the most important ones in King classification [4], because they allow a less aggressive surgical approach [16,6]. We found (Figure 3) that these curves behave in a very different way if compared to others, with a very high number of left directions and right shifts, and a prevalence of not sagittally shifted curves. This is true also looking at the results from the other side: almost 50% of left directions, right shift and not sagittally shifted curves are King 2. We don't know what this means, and further researches are needed to better understand the 3-DEMO classification, but this result gives a clue towards a possible clinical importance. On the contrary, we did not find any kind of correlation with Lenke classification in its single components, not even with lumbar and sagittal modifiers that in some way introduced a 3-D consideration: this should be better understood with future studies on clinical applications. Nevertheless recent papers questioned the validity of Lenke classification [17,18], as it happened before with King classification [7,8]: it seems that we are still searching for the best classification also in 2D, as it testifies a new proposal appeared recently while this paper was under review [17], even if all give some important clues to clinics.

The correlation between clinical and 3-DEMO parameters (Table 2), even if in many cases statistically significant, were very feeble (low RSquare), mainly with the exception of Cobb Index for Frontal Shift, but also Kyphosis (curiously more than Sagittal Index) for Sagittal Shift and Cobb frontal degrees for Phase.

Results about the 3-DEMO parameter Phase are peculiar, because it is not correlated with any other existing clinical classification, nor Ponseti or King or Lenke or Sagittal Configuration. This is a particularly relevant point, because in our mind the way in which frontal and sagittal curves (as we are used to see and think of in the spine) combine to cause Phase gives this parameter a real 3-D importance. The name [14] and the description we have just made demonstrate once again the fact that we think 2-D, but reality is 3-D: Phase is a true 3-D phenomenon, not scoliosis and kypho-lordosis as we are used to.

The modelling through a stepwise regression analysis allowed us to calculate 4 rather reliable models according to RSquare values. Interestingly, Direction and Phase have been better described using all parameters while, as awaited, Shifts required to radiographically analyze the Cobb degrees of the correspondent plane: the only exception was a light contribution of kyphosis on LL Shift. So, the "truest" 3-D parameters again appear to be Direction and Phase, confirming the already stated phenomenon that only an alteration of one of these parameters (even if both could be combined) can identify a scoliosis[14]: we could assume that the prevalence, in one patient, of Phase or Direction can represent different types of scoliosis.

## Conclusion

We have found some correlations between the 3-DEMO classificatory parameters and the classical radiographic classifications and measurements. These results support the hypothesis of a possible clinical significance for this classification, even if follow-up studies are needed to better understand these possible correlations and ultimately classification usefulness. Another study is needed to compare this classification with the 3D already existing [9,19] in order to understand how previously described classificatory items behave in the 3-DEMO environment.
